# Exercise, Prostaglandin E_2_, and Cardiometabolic Health: From Molecular Signaling to Systemic Adaptation

**DOI:** 10.3390/cells15141254

**Published:** 2026-07-12

**Authors:** Joseph Mannozzi, Mike M. Ravn Pedersen, Shaheen Y. Bhat, Timothy D. Bryson

**Affiliations:** 1 Cardiovascular Medicine, Henry Ford Health, Detroit, MI 48202, USA; 2 College of Human Medicine, Michigan State University, Lansing, MI 49503, USA; 3 Department of Physiology, Wayne State University School of Medicine, Detroit, MI 48202, USAshaheenyawarbhat@wayne.edu (S.Y.B.); 4 Department of Internal Medicine, Hypertension and Vascular Research Division, Henry Ford Health, Detroit, MI 49503, USA

**Keywords:** prostaglandin E_2_, exercise, cardiometabolic disease, inflammation, EP receptors, metabolic syndrome

## Abstract

Exercise is a cost-effective cornerstone therapy available to all for the treatment of cardiometabolic disorders, yet the molecular mediators linking physical activity to systemic metabolic and cardiovascular adaptations remain incompletely defined. Prostaglandin E_2_ (PGE_2_) is a bioactive lipid derived from arachidonic acid metabolism and has recently emerged as a critical regulator of inflammation, vascular function, mitochondrial remodeling, and glucose–lipid homeostasis. This review synthesizes current evidence on PGE_2_ biosynthesis and signaling in the context of exercise, with a focus on its broader role in cardiometabolic health and disease.

## 1. Cardiometabolic Disorders

### 1.1. Burden of Cardiometabolic Disorders and the Role of Exercise

Cardiometabolic disease (CMD) is an all-encompassing term that refers to comorbid disorders affiliated with traditional cardiovascular disease pathologies. These metabolic comorbid components include obesity, metabolic dysfunction-associated steatotic liver disease (MASLD), type 2 diabetes mellitus (T2DM), and metabolic syndrome. Importantly, these diseases are often coupled with ischemic heart disease and heart failure (HF). Moreover, the increasing prevalence of sedentary lifestyles, obesity, and metabolic comorbidities of cardiovascular disease has exacerbated the overall burden of CMD. Cardiovascular disease accounted for 915,973 deaths in the United States alone in 2023 [1] and presents a significant economic challenge in the coming years, as it is estimated to exceed $500 billion annually [1]. Furthermore, these estimates only take into consideration increases in traditional cardiovascular disease burden. Thus, these numbers are likely to further increase, especially with the observed increases in cardiometabolic pathologies, which typically present with poorer long-term outcomes and more expensive treatment regimens.

Pharmacological interventions for CMD are complicated by the complexity of various comorbidities associated with the different conditions that make up CMD. Addressing these conditions thus requires an understanding of the shared pathways between the various disorders and the development of novel, cost-effective therapies. One such therapy that has been proven over time to be effective for the treatment of cardiovascular and metabolic diseases, both in isolation and when combined in the context of CMD, is exercise. The term *exercise*, for the purpose of this review, encompasses any physical movement defined by structure and planned purpose (i.e., increasing physical fitness).

In general, exercise training is known to positively regulate insulin and glucose balance, improve body composition, and reduce waist circumference [2,3,4]. The magnitude of these effects, however, may vary depending upon the population, specific disease state, and the type of exercise being performed (i.e., aerobic, resistance, or combination training, discussed in more detail below). In addition to global metabolic benefits, exercise can also improve chronic inflammation, which can be an underlying factor that potentiates many cardiometabolic disorders [5]. Unfortunately, traditional markers of inflammation are too general to identify specific characteristic shifts in inflammatory status. Thus, a necessary remaining area of research in the field of CMD is to identify more precise biomarkers that can be used to specifically identify metabolic-related inflammation. As the healthcare system continues to be burdened by rising costs related to CMD, shifting the public’s focus towards prevention with lifestyle interventions like exercise should be a priority. At present, the recommended amount of exercise by the American Heart Association (AHA) for healthy individuals is 150–300 min per week. However, when it comes to obesity and CMD, the AHA recommends that the timeframe increase to 225–420 min of moderate-intensity aerobic activity per week for clinically meaningful weight loss, in combination with strength training and diet interventions [6]. Thus, even though exercise presents an excellent therapeutic intervention and prevention mechanism, if prevention is not achieved prior to the development of CMD, the burden on the patient to perform the prescribed exercise becomes greater and more difficult to maintain.

Besides weight loss, exercise is crucial for improving cardiorespiratory fitness (CRF); low CRF is a risk factor for CVD, CVD mortality, and all-cause mortality [7], especially in individuals with obesity and CMD. The Aerobics Center Longitudinal Study examined changes in fitness (metabolic equivalents; METs) and CVD mortality in 14,345 men. They identified that for every 1-met improvement, there was a 15% and 19% lower risk of all-cause and CVD mortality, respectively [7].

From a clinical standpoint, understanding the patients’ cardiometabolic risk factors can help guide the exercise regimen prescribed. To improve resting blood pressure, it has been shown that aerobic training, resistance training, or the combination of both have similar positive effects on resting systolic/diastolic blood pressure in adults, independent of obesity [8,9,10]. However, different exercise modalities (aerobic, resistance, or a combination) may induce distinct metabolic and inflammatory adaptations. Furthermore, the combination of aerobic and anaerobic resistance training has been shown to achieve moderate improvements in insulin sensitivity and glycemic control in type 2 diabetics [8], with an added benefit of improved lipid profiles [11]. Interestingly, for improving fat mass and CRF, aerobic training or a combination of aerobic and resistance training has a greater, more favorable effect than resistance training alone. For fat mass specifically, a combination approach provides the greatest benefits [12,13,14]. Aerobic training alone improves CRF in a dose-dependent fashion, whereby higher-intensity aerobic training increases CRF more than moderate-intensity training [15].

For patients currently suffering from CMD, understanding the molecular mechanisms of exercise-induced benefits is of utmost importance. Moreover, this would allow researchers to potentially develop novel exercise mimetics to supplement current pharmacological treatments for those patients who cannot participate in traditional exercise therapy due to the severity of comorbidities.

Understandably, the question of how exercise perpetuates improvements in CMD has been of considerable interest for researchers in recent years. Recent studies have shown that plasma from exercised mice, administered to older, cognitively deficient mice, mediated improved cognitive functions and reduced brain inflammation. This suggests that the effects of exercise are of a humoral nature (secreted factors termed *exerkines*), in addition to the known stimuli (increased cardiac output, increased vasoconstriction, shear stress, stretch receptor activation, etc.) [16].

A central challenge in the field of exercise mimetics is that not a single pathway but rather a system-level stimulus coordinates changes in skeletal muscle, cardiovascular function, metabolism, immunity, endocrine signaling, etc. Most studies examining exercise mimetics only target one or a few of the aforementioned pathways. This makes it particularly challenging to reproduce in translational studies. Moreover, at the human level, exercise intensity, sex, age, genetics, and disease state all play a role in shaping how these exercise-induced molecules may affect the system as a whole. There is considerable enthusiasm surrounding some key myokines like irisin, IL-15, apelin and others. However, the causal evidence remains heterogeneous as circulating levels are difficult to reliably measure and the mechanism of action for many of the potential candidates is poorly understood [17]. Given that PGE_2_ has both pro- and anti-inflammatory effects depending upon the tissue and stimulus, we propose a role for the bioactive lipid prostaglandin E_2_ in conferring exercise improvements (discussed in more detail in later sections).

### 1.2. Exercise-Induced Lipid Response

Ample evidence from the literature suggests that exercise training has positive effects on both the prevention and the treatment of cardiovascular disease. Importantly, one way that exercise reduces the burden of cardiovascular disease is through its impact on blood lipid concentrations. In general, exercise training reduces total triglyceride levels and increases high-density lipoprotein (HDL) cholesterol, while lowering low-density lipoprotein (LDL) cholesterol. The type of exercise is important, as a meta-analysis by Smart et al. [18] recently showed that when the types of exercise were set as covariates in the analysis, aerobic training significantly reduced total cholesterol, LDL, triglycerides, and very-low-density lipoprotein (VLDL) cholesterol. In contrast, resistance training only provided improvements in HDL. The effect of combined training was similar to that of aerobic training alone, via reductions in cholesterol, LDL, VLDL, and triglycerides, with a concomitant increase in HDL. Thus, the type, intensity, and duration of exercise are key factors that influence its beneficial effects.

Besides the beneficial changes in the species of lipoproteins, studies in both human and animal models have recorded changes in lipid mediators in response to exercise. Lipid mediators are bioactive signaling molecules that are derived from fatty acids to help coordinate the body’s response to acute and chronic exercise training. During and after exercise, the production of prostaglandins, leukotrienes, lipoxins, and other pro-resolving mediators (e.g., resolvins) helps to regulate inflammation, vascular tone, and adaptation. Among these mediators is prostaglandin E_2_, the focus of this review article (Table 1) [19,20,21,22,23,24].

## 2. Overview of Prostaglandin E_2_ Biology

### Biosynthesis and Metabolic Pathways

PGE_2_ is a bioactive lipid mediator belonging to the eicosanoid family, derived from arachidonic acid and synthesized through a tightly regulated, multi-step enzymatic process. It typically acts in a paracrine and/or an autocrine fashion and plays critical roles in inflammation, fever, pain sensitization, vascular tone regulation, and gastrointestinal protection. The biosynthesis of PGE_2_ occurs in most mammalian cell types and is typically initiated in response to physiological stimuli such as cytokines, growth factors, or mechanical injury [25]. The first and rate-limiting step in PGE_2_ synthesis involves the mobilization of arachidonic acid from membrane phospholipids. This cleavage is catalyzed primarily by cytosolic phospholipase A_2_ (cPLA_2_), which is activated by increases in intracellular calcium and phosphorylation via mitogen-activated protein kinases (MAPKs). Upon activation, cPLA_2_ selectively hydrolyzes the sn-2 position of glycerophospholipids, releasing free arachidonic acid into the cytosol [26]. This step is highly regulated, ensuring that prostaglandin synthesis is closely linked to cellular activation states. Once liberated, arachidonic acid is converted to prostaglandin H_2_ (PGH_2_) via the cyclooxygenase (COX) enzymes, which exist in two main isoforms: COX-1 and COX-2. COX-1 is constitutively expressed in many tissues and is responsible for basal prostaglandin production involved in homeostatic functions such as gastric mucosal protection and platelet aggregation. In contrast, COX-2 is an inducible enzyme, upregulated during inflammation in response to pro-inflammatory cytokines (e.g., IL-1β, TNF-α) [27,28] and other stimuli [29]. The COX enzymes catalyze a two-step reaction. First, arachidonic acid is oxygenated and cyclized to form prostaglandin G_2_ (PGG_2_), a hydroperoxide intermediate. This reaction requires molecular oxygen and involves the insertion of two oxygen molecules. Subsequently, the peroxidase activity of the same enzyme reduces PGG_2_ to PGH_2_. PGH_2_ serves as a common precursor for all major prostanoids, including prostacyclin (PGI_2_), thromboxane A_2_ (TXA_2_), and various prostaglandins such as PGE_2_.

The final step in PGE_2_ biosynthesis is the isomerization of PGH_2_ to PGE_2_, catalyzed by prostaglandin E synthases (PGES). There are three known isoforms of PGES. Microsomal PGES-1 (mPGES-1): This glutathione-dependent enzyme is inducible and functionally coupled to COX-2 [30]. It is the primary enzyme responsible for elevated PGE_2_ production during inflammatory responses. Microsomal PGES-2 (mPGES-2): Constitutively expressed and less well-characterized, it may contribute to both basal and inducible PGE_2_ synthesis. Cytosolic PGES (cPGES): A cytosolic form typically associated with COX-1 and responsible for housekeeping levels of PGE_2_. mPGES-1 is considered the dominant isoform during inflammation and is often upregulated alongside COX-2, creating a coordinated pathway for increased PGE_2_ production under pathological conditions.

The enzymes involved in PGE_2_ biosynthesis are strategically localized within the cell. cPLA_2_ translocates to perinuclear and endoplasmic reticulum membranes upon activation, where it releases arachidonic acid in proximity to COX enzymes. COX-1 and COX-2 are primarily membrane-bound enzymes located in the endoplasmic reticulum and nuclear envelope, facilitating efficient substrate channeling. Similarly, mPGES-1 is associated with these membranes, forming a functional complex with COX-2.

Regulation of PGE_2_ biosynthesis occurs at multiple levels, including enzyme expression (transcriptional control of COX-2 and mPGES-1), enzyme activation (phosphorylation of cPLA_2_), substrate availability, and feedback mechanisms. Nonsteroidal anti-inflammatory drugs (NSAIDs) exert their effects by inhibiting COX activity, thereby reducing PGE_2_ production [31].

PGE_2_ exerts its biological effects through four distinct G protein-coupled receptors (EP1–EP4), each linked to different intracellular signaling pathways [32,33,34]. Classically, EP2 and EP4 signal through G_αs_, activating adenylate cyclase and increasing cAMP levels within the cell. In contrast, EP3 signals through G_αi_ to inhibit adenylate cyclase. EP1 signals through G_αq_ to increase Ca^2+^ levels in the cell. The diversity of receptor subtypes allows PGE_2_ to mediate a wide range of physiological and pathological responses depending on the tissue context and receptor expression profile [35,36].

## 3. Exercise as a Mediator of PGE_2_ Production

### 3.1. Effect of Exercise on PGE_2_ Production

Several studies [37,38,39,40,41,42,43] have examined changes in prostaglandins, including PGE_2_ specifically, after exercise (summarized in Table 2). Trappe and colleagues reported that administration of the NSAIDs ibuprofen and acetaminophen at maximal doses can inhibit skeletal muscle protein synthesis after resistance exercise [37,38]. They followed up this study by showing that production of PGE_2_ in healthy young men was significantly attenuated after exercise when treated with acetaminophen specifically. The ibuprofen group had a trending reduction, but the data were not statistically significant. These results were confirmed when Naruse et al. examined the effects of low-dose aspirin on skeletal muscle PGE_2_ production after resistance training. In both men and women, low-dose aspirin inhibited muscle PGE_2_ production significantly compared to standard-dose aspirin [39]. Interestingly, the skeletal muscle of men was 60% more sensitive to the effects of aspirin. This warrants future studies on the COX/PGE_2_ pathway in the skeletal muscle of men and women and the broader impact of sex differences in the long-term consumption of NSAIDs, as inhibiting PGE_2_ would likely contribute to deleterious effects on exercise.

Taken together, these findings suggest that PGE_2_ is not simply a marker of the acute inflammatory response to exercise but rather an important mediator of skeletal muscle adaptation during training. The observations that NSAIDs attenuate exercise-induced PGE_2_ production indicate that COX-derived prostaglandin signaling may contribute to the anabolic response to exercise. Moreover, the differential sensitivity to aspirin observed between men and women raises the possibility that sex-specific regulation of the COX–PGE_2_ axis influences training adaptation and recovery. In the broader context of exercise physiology, these data support a model whereby transient increases in PGE_2_ following exercise serve as a physiological signal that promotes muscle repair and regeneration. In contrast, chronic pharmacological suppression of the pathway (e.g., NSAID use) likely blunts the beneficial adaptations to training. Thus, PGE_2_ represents a key mechanistic link between acute exercise and long-term structural/metabolic adaptations that improve overall muscle function and health.

Additional studies have examined the role of PGE_2_ and its four G-protein-coupled receptors on skeletal muscle and skeletal muscle stem cells (muscle satellite cells). These studies are described in more detail in Section 3.2, and while direct evidence may not be available, we cautiously explore how activity of the receptors may be linked to cardiometabolic disorders.

### 3.2. Role of EP Receptors in Skeletal Muscle

#### 3.2.1. EP1 Receptor (Ptger1)

The EP1 receptor is expressed at detectable, albeit relatively low, levels in human skeletal muscle and forms part of the broader COX–PGE_2_ signaling pathway that regulates muscle physiology. In a study from Liu et al., human muscle biopsy samples were obtained from the soleus (mainly type I fibers) and the vastus lateralis (>type 2 fibers than the soleus). Expression of EP1 and EP2 was not different between muscle types [40]. In a parallel experiment, samples from the same regions were also obtained from a sedentary group of young vs. old participants. EP1 (along with EP3 and EP4) has been shown to have reduced expression with aging, suggesting it may mediate muscle adaptation processes [40]. Unlike EP2 and EP4 (discussed below), EP1 has not been directly linked to muscle growth or regeneration pathways. Instead, it is thought to play a more modulatory role within the PGE_2_ signaling network. Based on its classically recognized signaling cascade, EP1 is hypothesized to contribute to Ca^2+^-dependent signaling, inflammation, and perhaps counter-regulation of anabolic pathways. This could subsequently promote endothelial dysfunction, vascular stiffness, insulin resistance, and ultimately an increased risk of cardiometabolic disease. However, direct evidence in skeletal muscle remains limited and incomplete and thus warrants further investigation.

#### 3.2.2. EP2 Receptor (Ptger2)

Muscle satellite cells are stem cells that are specific to skeletal muscle. These are adult stem cells that are situated between the muscle basal lamina and the sarcolemma. Upon injury, they are activated, enter the cell cycle (normally existing in a quiescent G_0_ state), undergo proliferation and differentiate into newly formed myofibers. They can also fuse with existing damaged myofibers for repair. These cells also play an important role in postnatal growth of muscle and hypertrophy, such as after exercise (via the Yap1/Taz-Thbs1-CD47 pathway) [44]. Their quiescence, however, is important as it prevents the loss and exhaustion of the cellular pool. Reports show that quiescence of muscle satellite cells is regulated by factors such as Wnt4 [45], N/M-cadherins [46], and Notch–Notch interactions [47]. Moreover, it was previously reported that the PGE_2_ EP2 receptor was upregulated in human muscle progenitors via Notch signaling mechanisms [48]. In a very recent study by Maruyama and colleagues, the authors elegantly show that EP2 is essential for the maintenance of muscle satellite cells, and its deficiency actually leads to their activation and loss, thereby impairing muscle regeneration. These actions of EP2 were via reduced phosphorylation of ERK1/2, similar to the actions of PTEN. Interestingly, the authors noted that EP2 and PTEN may have similar roles in the maintenance of the skeletal muscle pool, warranting further investigation into this novel relationship [49].

#### 3.2.3. EP3 Receptor (Ptger3)

EP3 receptor expression in skeletal muscle has been shown to be ~2-fold higher in the soleus (type 1 fiber) vs. the more glycolytic vastus lateralis muscle (type 2 > type 1), and, like EP1, was shown to decline ~30–33% with age [40]. Within skeletal muscle physiology, PGE_2_ signaling broadly regulates protein turnover and exercise adaptation. By analogy to its canonical signaling and effects in other tissues, EP3 is thought to bias responses toward reduced cAMP/PKA signaling, pro-inflammatory activity, and potentially catabolic or anti-anabolic effects, rather than regeneration or hypertrophy. Much like the EP1 receptor, direct mechanistic studies in skeletal muscle are lacking for EP3, but EP3 is strongly linked in other systems to inflammation [50], vascular contractility [51,52,53,54], and stress signaling [55,56,57,58] and mediates processes such as immune activation and cell stress responses via Gi/PLC pathways. Taken together, the available evidence supports a model in which EP3 acts as a modulatory or inhibitory arm of PGE_2_ signaling in skeletal muscle, contributing to the regulation of protein turnover and inflammatory tone, but with comparatively sparse direct evidence defining its specific functional role relative to the better-characterized EP2/EP4 pathways.

#### 3.2.4. EP4 Receptor (Ptger4)

The EP4 receptor is one of the most well-characterized PGE_2_ receptors in skeletal muscle and plays a central role in muscle regeneration [43], growth [41], and metabolic regulation [59]. EP4 is primarily a Gαs-coupled GPCR that stimulates adenylate cyclase, increasing cAMP and activating downstream PKA/CREB signaling pathways, affecting target genes such as Nurr1 (*Nr4a2*) [41] and *Orai1* [60], which share common functions in immune activation and metabolism. Similarly, a very recent report by Jiang et al. showed that in *migrasomes* (these are membranous organelles released from migrating cells), upregulation of PGE_2_ induced an SPP1+ (secreted phosphoprotein 1) macrophage phenotype [61]. The authors did not explore the specific PGE_2_ receptor mediating this response; however, this agrees with previously reported roles for the EP4 receptor in the cancer literature [62,63,64]. In skeletal muscle, EP4 signaling has been shown to be essential for muscle satellite cell function, where PGE_2_ acting through EP4 promotes myoblast proliferation, satellite cell expansion, and efficient regeneration after injury, with genetic deletion of EP4 impairing regeneration and reducing muscle strength. In the study from Wang et al., the authors generated a satellite cell-specific EP4 knockout and showed an accelerated aging phenotype characterized by atrophy and loss of strength [43]. Mechanistically, EP4-mediated cAMP signaling aligns with broader evidence that cAMP pathways drive hypertrophy, regeneration, and metabolic adaptation in skeletal muscle, including the regulation of fiber size and oxidative phenotype. Importantly, EP4 signaling is also altered with aging, with reduced EP4 activity in aged muscle stem cells contributing to impaired regeneration. Moreover, it has been reported that PGE_2_ levels themselves are reduced with aging due to increased expression of 15-hydroxylprostaglandin dehydrogenase (15-PGDH), an enzyme that degrades prostaglandins [42], appropriately termed a *gerozyme* [65]. In contrast, restoring PGE_2_–EP4 signaling improves muscle repair and function [41]. From a cardiometabolic perspective, EP4 exerts dual and context-dependent effects: on one hand, it can be beneficial by supporting tissue repair and suppressing inflammation in some settings (e.g., improving insulin sensitivity via anti-inflammatory actions in obesity models); on the other hand, emerging evidence in skeletal muscle cells indicates that EP4 activation can impair insulin signaling and glucose uptake via NF-κB-mediated inflammatory pathways, contributing to lipid-induced insulin resistance. Together, these findings support a model in which EP4 is a key anabolic and regenerative receptor in skeletal muscle, but when dysregulated, particularly under conditions of chronic inflammation, lipid overload, or aging, it may also contribute to metabolic dysfunction and insulin resistance, linking muscle biology to broader cardiometabolic disease risk.

## 4. PGE_2_ Response to Exercise in Adipose Tissue

PGE_2_ has emerged as an important regulator of adipose tissue physiology, particularly in the context of lipolysis, inflammation, and metabolic adaptation. A key feature of PGE_2_ signaling in adipose tissue is its tight coupling to lipid mobilization. Although relatively few studies have directly examined PGE_2_ responses in adipose tissue following acute or chronic exercise, a substantial body of work in adipocyte biology and related physiological conditions (i.e., fasting, lipolysis, COX-2 activation) provides mechanistic insights that may be relevant to exercise adaptation. Therefore, much of the following discussion should be interpreted as hypothesis-generating and based on indirect evidence rather than direct demonstrations of exercise-induced adipose PGE_2_ signaling.

For example, lipolytic stimulation of adipocytes has been shown to increase PGE_2_ production via activation of classical PLA2 and COX pathways. This indicates that PGE_2_ is generated as part of the adipocyte response to triglyceride breakdown. This is particularly relevant to exercise, where catecholamine-driven lipolysis is a well-known dominant metabolic process. However, direct evidence that exercise-induced lipolysis increases adipose tissue PGE_2_ production in humans remains limited.

PGE_2_ signaling through its EP receptors, especially EP4, can modulate basal lipolysis and interact with insulin signaling, suggesting a role in fine-tuning fatty acid release during dynamic energy demand. However, this proposed role during exercise is currently inferred from adipocyte and pharmacological studies rather than direct exercise experiments. Consistent with this, exogenous PGE_2_ has been shown to inhibit β-adrenergic-stimulated lipolysis in adipocytes, further supporting a feedback limit on excessive lipid mobilization [66,67,68].

In addition to its metabolic regulation, PGE_2_ plays a significant role in adipose immune function. Adipocyte-derived PGE_2_ acts as a chemoattractant and signaling mediator that promotes macrophage recruitment during lipolysis, linking lipid mobilization to immune cell trafficking. Importantly, this effect appears to be largely anti-inflammatory or regulatory rather than overtly pro-inflammatory, consistent with other recently discovered functions of the EP4 receptor [66,69,70]. Indeed, PGE_2_ signaling through EP4 receptors suppresses chemokine production, dampens inflammatory gene expression in adipose tissue and can reduce the expression of cytokines such as IL-6 and MCP-1 in human adipose explants [71]. These findings align with the well-established anti-inflammatory effects of regular exercise in adipose tissue and raise the possibility that PGE_2_ contributes to exercise-induced improvements in adipose immune tone. However, direct studies measuring adipose PGE_2_ signaling alongside exercise-induced immune remodeling are currently lacking. Moreover, any contribution of PGE_2_ to exercise-mediated adipose immune adaptations remains speculative at present.

PGE_2_ also influences adipose tissue remodeling and thermogenic programming, which are especially important in the context of exercise and obesity. Adult humans with detectable levels of brown fat have a reduced incidence of hypertension [72]. In both human and experimental models, PGE_2_ promotes the expression of brown/beige adipocyte markers, including uncoupling protein 1 (UCP1) and PR domain zinc finger protein 16 (PRDM16), indicating a role in adipose browning and adaptive thermogenesis. This is particularly relevant to exercise physiology, as endurance training is known to enhance mitochondrial capacity and promote beige adipocyte formation. Whether PGE_2_ directly mediates these exercise-induced adaptations in adipose tissue has not been established. Very recently, Koenen et al. elegantly showed that in adipocyte-specific *Prdm16* knockout mice, characterized by a loss of beige adipocyte identity, there was marked remodeling of perivascular adipose tissue, increased vascular reactivity, and elevated blood pressure [73]. Furthermore, PGE_2_ production appears to restrain adipogenesis and adipose tissue expansion. In contrast, loss of COX-2 signaling increases adipocyte size and number, whereas PGE_2_ suppresses adipogenesis through PKA-dependent pathways (e.g., EP4 signaling) and reduces fat accumulation. A timely, relevant study was performed by Jingyi Li et al. [74] using a glucagon-like peptide-1 receptor agonist (GLP-1RA), Liraglutide. The authors showed that Liraglutide reduced body weight, improved metabolic insulin resistance and increased the expression of browning-related markers like PGC1α, UCP1, and ATGL. Importantly, COX-2 signaling and prostaglandin levels were increased in subcutaneous tissues. COX-2 inhibition abolished the effects of Liraglutide. These results confirmed that targeting the COX-2 pathway is important to consider when treating obese patients with GLP-1RAs. Together, these findings position PGE_2_ as a regulator not only of acute metabolism but also of long-term tissue remodeling [74,75]. However, definitive evidence supporting a similar role during exercise-induced adipose remodeling remains limited.

As mentioned previously, exercise is a key mechanism for reducing obesity, insulin resistance, inflammation, and ultimately cardiometabolic syndrome. Although direct PGE_2_ measurements in adipose tissue following exercise are scarce, studies using related physiological paradigms such as fasting, lipolysis-inducing interventions, genetic models, and pharmacological manipulation provide a conceptual framework for understanding how PGE_2_ may participate in exercise adaptation. Importantly, these studies do not establish that exercise elicits identical PGE_2_-dependent mechanisms in adipose tissue. The beneficial effects of PGE_2_ on immune and metabolic adaptations observed during intermittent fasting share several physiological features with endurance exercise. However, extrapolation between these conditions should be interpreted cautiously because the underlying signaling responses may not be identical. Future studies directly measuring adipose PGE_2_ responses to acute and chronic exercise in humans will be important to confirm and expand upon this framework [66,67,68,71].

## 5. Preclinical Large-Animal Evaluations of PGE_2_ and Implications for Sensitization of Autonomic Response Mechanisms in Humans

PGE_2_ has been examined with regard to cardiovascular function in a variety of large-animal model systems, including leporine, feline, canine, porcine, ovine, and bovine models [76,77,78,79,80]. Although PGE_2_ has been evaluated comparatively across multiple models, conclusions vary regarding cardiovascular function outcomes. Specifically, there are variations in results related to how PGE_2_ function is altered depending on the study design and the specific portion of the prostaglandin pathway being evaluated [76,77,79,81,82,83]. For instance, early studies in large animals have shown that prostaglandins (PGE_2_ specifically) can modulate autonomic responses, contribute to ventricular remodeling following a myocardial infarction, and participate in ischemia–reperfusion preconditioning [77,84,85,86,87,88]. Studies evaluating baroreflex activity and intracoronary PGE_2_ infusion have shown that PGE_2_ is capable of impairing baroreflex function independently of chemoreflex activity, and this is likely driven by cardiac c-fiber afferent activity [76]. Coincidentally, inhibition of the COX pathway with the COX inhibitor indomethacin was shown to blunt cardiac afferent responses to bradykinin in dogs with congestive heart failure [88]. Together, these results imply that PGE_2_ activity increases cardiac afferent activity and impairs baroreflex activity [89,90,91,92]. Beyond direct autonomic alterations, studies utilizing large animals have also shown that the inhibition of enzymes responsible for PGE_2_ synthesis, specifically COX-2, leads to exaggerated vasoconstriction, impaired response to myocardial infarction, and abolition of ischemia–reperfusion preconditioning. Although studies utilizing exercise per se are limited, these aforementioned studies highlight the potentially beneficial impact of PGE_2_ signaling in response to exercise (e.g., vasoconstriction) and myocardial injury [77,78,79,87].

In contrast to cardiac function, the skeletal muscle response to PGE_2_ in preclinical models is far less understood. Current evidence is largely limited to the potential role of PGE_2_ signaling in skeletal muscle afferent nerves, as well as its release and downstream systemic effects. Even within the studies on afferent activity, preclinical relevance remains unclear, in part because there is no clearly defined stimulatory pathway by which prostaglandins can directly regulate autonomic function. Nevertheless, prostaglandins remain an important topic of discussion regarding autonomic function, particularly in the context of cardiometabolic disease.

Studies evaluating cardiac sympathetic afferent activity have identified a potential role for PGE_2_ and/or COX-2 inhibition as described above, illustrating the potent impact of prostaglandin activity on cardiac afferent activation within the heart and cardiac circulation as well as its robust impact on baroreflex function [77,82,83,93,94,95,96]. However, it remains unclear whether prostaglandins act within the cardiac afferent system directly or through other pathways (i.e., skeletal muscle, renal afferents). Moreover, given the limited studies of PGE_2_ and altered autonomic activity, it is possible that the effects are a result of the actions of prostacyclin (or another COX-1/2-derived prostanoid). Based on the current evidence, it is likely that COX-derived mediators act primarily to sensitize or desensitize neural responses to more potent autonomic mediators, as opposed to being direct stimulators of autonomic activity themselves. However, their innate signaling capacity has yet to be investigated. Regarding indirect alterations in autonomic function, relatively recent studies of prostaglandins have shown that they can sensitize or enhance responses mediated by common receptors (TRPV1, P2X3, and ASIC) located on type III and IV unmyelinated afferent fibers, which are known to contribute to both pain signaling and cardiovascular response patterns [97,98,99,100,101,102]. This capacity to sensitize afferent receptor subtypes is especially important as several cardiovascular and metabolic conditions result in elevated inflammatory responses, in which PGE_2_ is likely involved. In the context of exercise and exercise capacity in disease, the potential effects of PGE_2_ expression are especially important. For instance, studies have shown that skeletal muscle-mediated cardiovascular responses via various afferent receptor subtypes (i.e., P2X3, TRPV1, and ASIC) are impaired and/or exaggerated in various disease pathologies such as diabetes, heart failure, hypertension, obesity, and others [96,103,104,105,106,107]. Given the inflammatory nature of these conditions, PGE_2_ signaling could be a mechanism that accounts for variations in autonomic response patterns from skeletal muscle during exercise, although direct evidence for this is currently lacking. In hypertension, for example, cardiac performance increases along with systemic vasoconstriction [108]. Additionally, in heart failure, cardiac performance improves minimally, yet vasoconstriction is profoundly exaggerated [109]. In the context of diabetes, pressor responses are exaggerated during exercise along with limited cardiac improvements relative to the other conditions, which is unexpected given the lack of existing hypertension [110]. Overall, the variations in the response patterns and severity of perfusion limitation in disease induced by autonomic afferent signaling may be modifiable via PGE_2_ and/or COX-2 modulation.

Studies in humans with heart failure have shown that when exercise training is not limited by intolerance and can be used as an adjunct therapy, mediators of autonomic dysfunction may normalize, which correlates with reductions in COX-2 pathway activation [98]. Subsequent studies have observed that afferent expression of both initiators of autonomic signaling (PGE_2_ and/or COX-2) is upregulated in individuals with reduced exercise capacity due to training status and disease [111]. However, this is not direct evidence in a conscious model, but the potential of prostaglandins as amplifiers and/or sensitizers of afferent signaling is plausible. Furthermore, the work in large animals and humans thus far has mainly centered on neurogenic mechanisms. Investigations on the effects of PGE_2_ and/or COX-2 in skeletal muscle itself regarding exercise capacity are limited. Thus, the unanswered questions regarding PGE_2_ signaling and autonomic function provide significant justification for further studies and evaluation as a therapeutic target for exercise intolerance.

## 6. Conclusions and Perspectives

The purpose of this review is to highlight the complex and context-dependent role of PGE_2_ in cardiovascular disease, exercise, and, importantly, its broader implications for cardiometabolic disease. The evidence presented here suggests that PGE_2_ signaling can play a protective and adaptive role in cardiovascular function (depending on which receptor subtype is activated and in which tissue), obesity, and inflammation, and can contribute to the modulation of autonomic responses. At the same time, inhibition of PGE_2_-producing pathways can exacerbate vasoconstriction and impair recovery following cardiac injury, highlighting its physiological importance. Preclinically, its role in skeletal muscle and afferent signaling remains less clearly defined, with current data suggesting that prostaglandins may act primarily as sensitizers of neural pathways rather than direct regulators of autonomic output. This concept is particularly relevant to cardiometabolic disease, where dysregulated autonomic function, chronic inflammation, and impaired exercise capacity intersect. The observed upregulation of COX-2 and prostaglandin-sensitive signaling pathways in individuals with reduced exercise tolerance suggests that altered PGE_2_ signaling may contribute to the autonomic imbalance and reduced cardiovascular adaptability characteristic of cardiometabolic conditions. Thus, PGE_2_ may represent a key intermediary linking inflammatory signaling, neural control, and cardiovascular dysfunction within the broader cardiometabolic disease framework, highlighting its potential as both a biomarker and therapeutic target.

## Figures and Tables

**Table 1:** Studies examining lipids in response to exercise.

Study	Design	Cohort	Lipids Measured	Key Findings
Jurado-Fasoli et al., 2022 [19]	12-week RCT	Middle-aged sedentary adults	Oxylipins (AA-derived: 12-HETE, 15-HETE; DHA/EPA-derived: resolvin precursors); Endocannabinoids, eCB and eCB-like molecules	No significant change in fasting plasma levels of Omega-3/6 oxylipins, nor any change in eCBs or eCB-like molecules.
Nieman et al., 2019 [20]	Crossover cycling trial	Endurance-trained cyclists	CYP oxylipins (EpOMEs, DiHOMEs), LOX oxylipins (HETEs, HODEs)	Prolonged exercise elevated 43 out of 45 metabolites analyzed; carbohydrate intake attenuates the increase.
van Doorslaer et al., 2023 [21]	Acute + training intervention	Healthy & prediabetic adults	Endocannabinoids (AEA, 2-AG, OEA, PEA); CB1/CB2 protein in muscle	Exercise modality-specific changes in ECs: Increase after endurance exercise, decrease after resistance training.
Zemski-Berry et al., 2025 [22]	Exercise + weight loss	Adults with obesity	DAG species (C16:0/18:1), sphingolipids (ceramides: Cer d18:1/16:0), triglycerides	Subcellular lipid redistribution linked to improved insulin sensitivity with weight loss.
Fabre et al., 2025 [23]	Mechanistic (animal + in vitro)	Mouse + muscle cells	Prostaglandins (PGD2, 15Δ-PGJ2), SPMs (Protectin D1)	Lipid mediator class switching controls myogenesis and regeneration.
Liu et al., 2025 (Review) [24]	Training/mechanistic	Human/animal muscle	Lipid droplet proteins (PLIN2, PLIN5) associated with triglyceride pools	Exercise remodels lipid droplets and enhances mitochondrial coupling.

Representative human and animal studies examining changes in bioactive lipid mediators in response to acute and chronic exercise. The table summarizes study design, participant characteristics, lipid classes measured (including oxylipins, endocannabinoids, sphingolipids, and prostaglandins), and key findings. Studies include both circulating (plasma/serum) lipid measurements and skeletal muscle lipidomic or signaling responses, highlighting systemic and tissue-specific adaptations to exercise.

**Table 2:** Studies examining prostaglandins in response to exercise.

Study	Design	Cohort	Compartment	Measurement	Key Findings on PGE_2_
Trappe et al., 2001 [37]	Acute eccentric resistance exercise ± NSAIDs	Healthy young adults	Skeletal muscle	Muscle biopsy (PGE_2_, PGF2α)	Eccentric exercise increased skeletal muscle PGE_2_ production
Trappe et al., 2002 [38]	Acute resistance exercise ± NSAIDs	Young adults	Skeletal muscle	Muscle prostaglandin signaling	Inhibition of PGE_2_ alters muscle remodeling signaling after exercise
Naruse et al., 2021 [39]	Acute resistance exercise + aspirin	Healthy adults (men vs. women)	Skeletal muscle	Ex vivo muscle PGE_2_ production	PGE_2_ reduced with aspirin; no significant change at 3.5 h post-exercise
Liu et al., 2016 [40]	Cross-sectional	Young and older adults (men vs. women)	Skeletal muscle	COX enzymes, PGE_2_ synthases, EP receptors	Confirms muscle capacity for PGE_2_ production relevant to adaptation and muscle fiber type
Ho et al., 2017 [41]	Muscle injury model	Mouse	Skeletal muscle	Satellite cell signaling	PGE_2_ required for regeneration after contraction-related injury
Palla et al., 2020 [42]	Pharmacologic elevation of PGE_2_ using 15-PGDH	Aged mice	Skeletal muscle	PGE_2_ signaling	Elevated PGE_2_ restores muscle mass and exercise capacity
Wang et al., 2025 [43]	Exercise + PGE_2_ treatment	Aged mice	Skeletal muscle	Stem cell signaling	PGE_2_ enhances exercise-induced regeneration

Studies examining prostaglandin E_2_ (PGE_2_) responses to exercise across biological compartments. The table distinguishes measurements in skeletal muscle, plasma, and urine, including direct quantification of PGE_2_ or its metabolites (e.g., urinary PGE-M). The majority of evidence derives from skeletal muscle biopsy or ex vivo production assays, whereas urinary metabolites reflect systemic prostaglandin turnover and plasma measurements remain limited. This compartmental stratification highlights differences between local (muscle) and systemic PGE_2_ responses to various exercise training paradigms.

## Data Availability

No new data were created or analyzed in this study.
